# Outcome of corticosteroid administration in autoimmune pulmonary alveolar proteinosis: a retrospective cohort study

**DOI:** 10.1186/s12890-015-0085-0

**Published:** 2015-08-12

**Authors:** Keiichi Akasaka, Takahiro Tanaka, Nobutaka Kitamura, Shinya Ohkouchi, Ryushi Tazawa, Toshinori Takada, Toshio Ichiwata, Etsuro Yamaguchi, Masaki Hirose, Toru Arai, Kentaro Nakano, Takahito Nei, Haruyuki Ishii, Tomohiro Handa, Yoshikazu Inoue, Koh Nakata

**Affiliations:** Bioscience Medical Research Center, Niigata University Medical and Dental Hospital, 1-754 Asahimachi-dori, Niigata, 951-8520 Japan; Uonuma Institute of Community Medicine, Niigata University Medical and Dental Hospital, Niigata, Japan; Department of Respiratory Medicine, Tohoku University Graduate school of Medicine, Miyagi, Japan; Department of Pulmonary Medicine, Tokyo Medical School, Tokyo, Japan; Department of Respiratory and Allergy Medicine, Aichi Medical School, Aichi, Japan; Clinical Research Center, NHO Kinki-Chuo Chest Medical Center, Osaka, Japan; Department of Respiratory Medicine, Dokkyo Medical University Koshigaya Hospital, Saitama, Japan; Department of Respiratory Medicine, Nippon Medical University of Medicine, Tokyo, Japan; Department of Respiratory Medicine, Kyorin University School of Medicine, Tokyo, Japan; Department of Respiratory Medicine, Kyoto University Hospital, Kyoto, Japan

## Abstract

**Background:**

Although no report has demonstrated the efficacy of corticosteroid therapy for autoimmune pulmonary alveolar proteinosis (aPAP), we sometimes encounter patients who have received this therapy for various reasons. However, as corticosteroids can suppress alveolar macrophage function, corticosteroid therapy might worsen disease severity and increase the risk of infections.

**Methods:**

For this retrospective cohort study, we sent a screening form to 165 institutions asking for information on aPAP patients treated with corticosteroids. Of the resulting 45 patients screened, 31 were enrolled in this study. We collected demographic data and information about corticosteroid treatment period, dose, disease severity score (DSS) over the treatment period, and complications.

**Results:**

DSS deteriorated during corticosteroid therapy in 23 cases (74.1 %) and the estimated overall cumulative worsening rate was 80.8 % for the total observation period. The worsening rate was significantly higher in patients treated with high-dose prednisolone (>18.9 mg/day, n = 16) than treated with low-dose prednisolone (≤18.9 mg/day, n = 15) divided by median daily dose (*p* < 0.02). Of patients with worsening, one died of disseminated aspergillosis and another of respiratory failure. Infections newly emerged in 6 cases during corticosteroid therapy (*p* < 0.05). Median serum granulocyte/macrophage colony-stimulating factor (GM-CSF) autoantibody levels were similar to previously reported data in a large cohort study.

**Conclusion:**

The results demonstrate that corticosteroid therapy may worsen DSS of aPAP, increasing the risk for infections.

**Electronic supplementary material:**

The online version of this article (doi:10.1186/s12890-015-0085-0) contains supplementary material, which is available to authorized users.

## Background

Pulmonary alveolar proteinosis (PAP) is a rare lung disease in which surfactant materials accumulate abnormally in the terminal bronchioli and alveoli [1, 2]. According to the etiology, PAP can be classified into three forms; hereditary, secondary, and autoimmune PAP [2]. Hereditary PAP are caused by some gene abnormality, for example, granulocyte/macrophage colony-stimulating factor (GM-CSF) receptor α or β insufficiency, in which neither serum GM-CSF autoantibodies nor underlying diseases are confirmed [3–5]. Secondary PAP develops secondary to some underlying diseases that presumably impair surfactant clearance because of abnormal numbers and functions of alveolar macrophages [6]. In patients with autoimmune PAP (aPAP), comprising 90 % of all PAP cases, GM-CSF autoantibody (GMAb) is excessively produced [2, 7, 8]. GMAb interferes with GM-CSF signaling in alveolar macrophages, causing maturation arrest and dysfunction and thus impairing surfactant catabolism [9, 10].

Currently, the standard therapy for PAP is whole-lung lavage [11–13]. Alternatively, since the mouse defective in GM-CSF production develops PAP, GM-CSF administration by injections or inhalation has been carried out with variable success [14–18].

As corticosteroid administration has been applied to treat a number of autoimmune diseases, it is a reasonable assumption that corticosteroid therapy might also be effective treatment modality for aPAP. Evidence supporting this notion includes observations that low-dose corticosteroids stabilize the cell membrane and maintain cell survival, that moderate-dose corticosteroids suppress the production of immunoglobulin by B cells, and that high-dose corticosteroids induce apoptosis of naive B cells and thus further suppress the production of immunoglobulins [19–21]. On the other hand, corticosteroids might inhibit phagocytosis and catabolism by alveolar macrophages by suppressing their cytokine/chemokine production, possibly leading to a deterioration in aPAP disease state [22–24]. corticosteroid therapy might also suppress the bactericidal activity of granulocytes and macrophages, resulting in increased susceptibility to infections [25, 26]. Furthermore, it might worsen aPAP due to acceleration of surfactant production in alveolar type II cells [27]. Thus, corticosteroid therapy for autoimmune PAP has been controversial. Based on a viewpoint that corticosteroid may suppress the production of autoantibody, its administration might be helpful for the treatment of aPAP. In contrast, based on another viewpoint that corticosteroid may suppress the alveolar macrophage functions, its administration might be harmful [28, 29]. To settle this controversy, we investigated retrospectively the prognosis of patients with autoimmune PAP who had been treated with corticosteroids.

## Methods

### Subject and study design

**Fig. 1:**
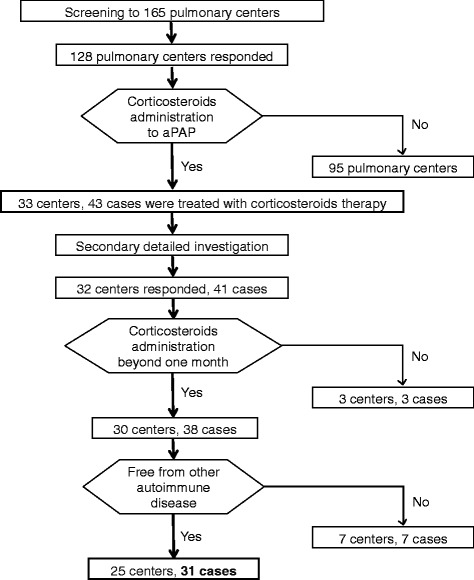
Profile of study cohort. aPAP: autoimmune pulmonary alveolar proteinosis

For the first screening, we sent a screening form to 165 major pulmonary centers in Japan since November 21th 2013 to November 20th 2014, inquiring about their experience with corticosteroid-administrated aPAP cases (Fig. 1). The screening form was sent mainly by e-mail and partially by mail. We sent additional investigation by e-mail when the answers were inadequate. We also used telephone if needed. Cases that met the following criteria were enrolled in this study: 1) with a definite diagnosis of aPAP, 2) treated with corticosteroid therapy for more than one month, and 3) without complication by other autoimmune diseases. Diagnosis of aPAP was based on cytological analysis of BALF or pulmonary histopathological findings, with both HRCT appearance and positive serum GMAb levels (≥1.0 μg/mL) [30, 31]. In total, 128 centers (78 %) responded to the screening, with 33 centers having experienced a total of 43 aPAP cases that were treated with corticosteroid therapy. To these centers, we sent a secondary investigation on patient details to the treating physicians and we received answers on 41 cases from 32 centers. Seven cases from 7 centers were excluded for complications by other autoimmune diseases and 3 cases for being treated with corticosteroid therapy for less than one month.

The following information was collected in the secondary investigation: age, gender, date of diagnosis, symptoms, present illness, past history, complicating diseases, smoking status, and history of dust exposure. Also requested was information on corticosteroid therapy, i.e., treatment duration, corticosteroid type, dosage, recorded reason for prescribing corticosteroid therapy, and presence of other accompanying therapies. In addition, we also collected data on disease severity score (DSS), and serum Krebs von den Lungen-6 (KL-6) levels at just before and various periods during corticosteroid therapy, and 6 and 12 months after the discontinuation of corticosteroid therapy.

**Fig. 2:**
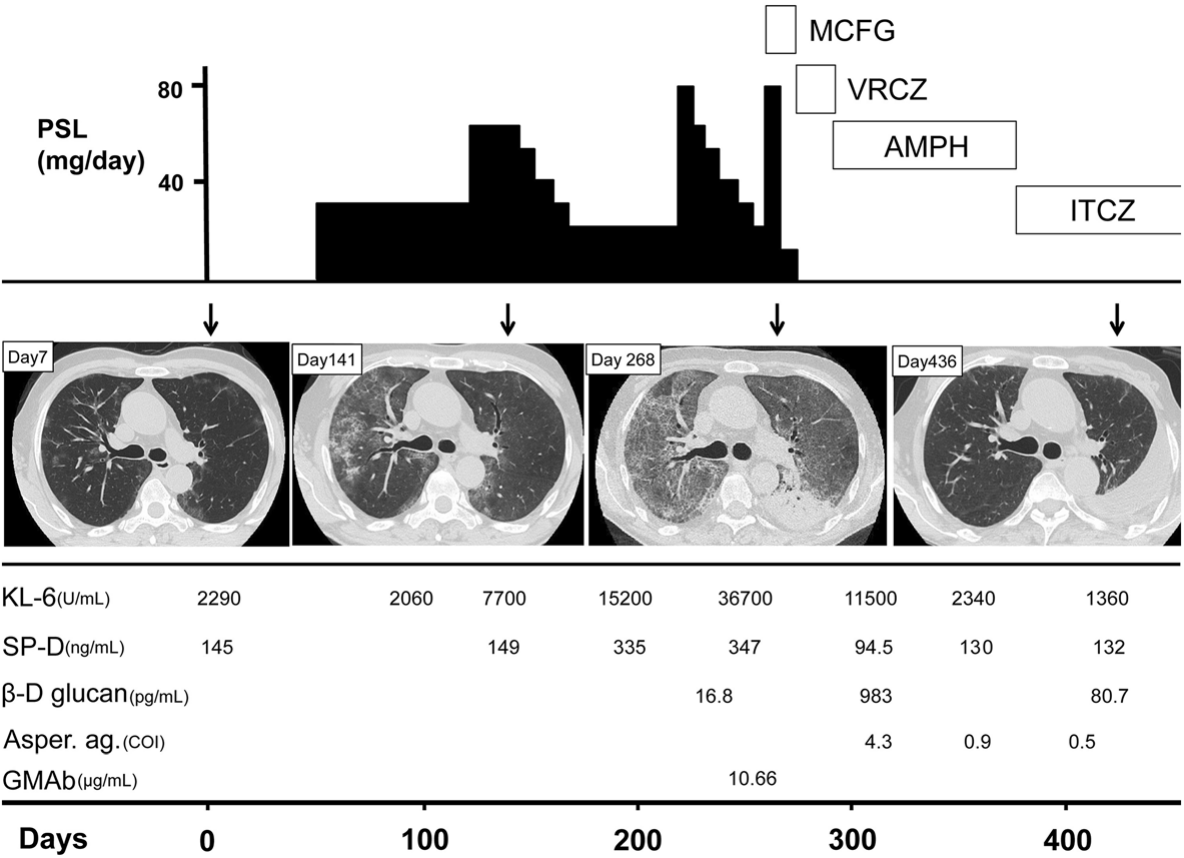
A typical clinical course of a case of autoimmune pulmonary alveolar proteinosis treated with corticosteroid administration. The upper column indicates schematic dose of the administered prednisolone (PSL) and antifungal agents each day. The middle column indicates the time course of chest HRCT appearance (Days after the first visit were shown by arrays). The lower column indicates serum levels of Krebs von den Lungen-6 (KL-6), surfactant protein D (SP-D), β-D glucan, *Aspergillus* antigen (Asper. ag.) and granulocyte/macrophage colony-stimulating factor autoantibody (GMAb). MCFG: Micafungin; VRCZ: Voriconazole; AMPH: amphotericin B; ITCZ: Itraconazole

The Institutional Review Board of Niigata University approved this study (approval no. 1792) and all pulmonary physicians agreed to collaborate with us. Data of patients were anonymously handled in a linkable manner. We obtained written informed consent from the represented case of shown in Fig. 2. The study protocol was designed according to The Ethical Guidelines 2008 for Clinical Studies by the Japanese Ministry of Health, Labour, and Welfare.

### Disease severity scoring

As the primary endpoint of this study, DSS was determined from the data obtained by investigation based on the following definition, and was entered into a database at each time point for every subject. DSS at the start of corticosteroid therapy and various time points were determined as described previously [8]. Briefly, DSS was determined based on the presence of symptoms and degree of reduction in PaO_2_ under room air in the supine position. The categories included: DSS 1 = no symptoms and PaO_2_ ≥ 70 mmHg; DSS 2 = symptomatic and PaO_2_ ≥ 70 mmHg; DSS 3 = 60 mmHg ≤ PaO_2_ < 70 mmHg; DSS 4 = 50 mmHg ≤ PaO_2_ < 60 mmHg; and DSS 5 = PaO_2_ < 50 mmHg. Qualifying symptoms included dyspnea or cough related to PAP. In subjects for whom PaO_2_ was unavailable, oxygen saturation was used to estimate the PaO_2_ as follows: oxygen saturation values of 94 %, 90 %, and 85 % were used as the values representing PaO_2_ cut-off values of 70, 60, and 50 mmHg, respectively.

As the percent diffusing capacity of the lung for carbon monoxide (%DLco), which is known to be a disease marker of PAP, had linear correlation with DSS through DSS of 1 to 5, we considered that any increase in DSS reflected the progression of disease [8]. Therefore, in this study, we defined “worsening” as any increase in the DSS.

### Measurement of GM-CSF autoantibodies

The GM-CSF autoantibody concentrations in the serum were measured using direct ELISA as described previously [30, 31].

### Statistical analysis

Differences between two groups were compared using the Wilcoxon’s rank-sum test for the continuous variables, and differences in proportions were evaluated using Chi-squared test or Fisher’s exact test. Differences in paired-proportions for frequencies of pulmonary infections before and after corticosteroid therapy were compared using McNemar’s test. Relationships between DSS and other parameters were analyzed using Spearman’s rank-order correlation. Over all cumulative worsening rate from the date at beginning of corticosteroid therapy until the date of worsening of DSS was estimated according to the Kaplan-Meier method. The Kaplan-Meier method is calculated by multiplying the probability of the worsening event time with the probabilities of worsening all the previous event times. A log-rank test was used to compare the difference in the cumulative probabilities between two groups. Variables that achieved statistical significance in the log-rank test were subsequently included in a multivariate analysis using a stepwise Cox regression procedure. *P* < 0.05 was considered to be statistically significant (two-sided). Analyses were performed using JMP 10.0 (SAS Institute Inc., Cary, NC, USA) and R-version 3.1.2 (R Foundation for Statistical Computing, Vienna, Austria).

## Results

### A representative case report

First, we report a typical case that received corticosteroid therapy for 216 days (Fig. 2). A 48-year-old male who had occasional dry cough was found to have an abnormality in chest X-ray during a medical health check and was directed to visit a hospital for intensive examination. HRCT appearance demonstrated multifocal faint ground-glass opacities (GGO). Due to the HRCT appearance and serum KL-6 levels that had been elevated for 2 months, the physician decided to start 30 mg of prednisolone (PSL) orally without pathological/cytological examination of the lung. After 4 months, HRCT appearance worsened and serum KL-6 levels were further elevated. The patient then underwent bolus methylprednisolone administration (mPSL; 1000 mg/day) for 3 days, followed by oral PSL medication tapering from 80 to 20 mg over 40 days. During the next 9 months, bolus mPSL administration was repeated twice without improvement in GGO, followed by emergence of consolidation in the lower left lung on HRCT with elevation of serum β-D glucan levels and *Aspergillus* antigen titer. GGO findings on HRCT and high serum KL-6 levels let the physician to suspect PAP, a diagnosis that was later confirmed by positive GM-CSF antibody in serum and characteristic BAL fluid appearance. Corticosteroid therapy was discontinued and administration of anti-fungal antibiotics was initiated. The consolidation on HRCT improved, with an accompanying reduction in β-D glucan and *Aspergillus* antigen levels in the serum. Importantly, GGO on HRCT remarkably improved within 3 months after discontinuation of corticosteroid therapy.

### Retrospective cohort

#### Demographic and clinical findings for study subjects

Demographic data of 31 study subjects obtained at the start of corticosteroid therapy since 2003 to 2014 and at the diagnosis of PAP are shown in Table 1. In 29 cases, corticosteroid therapy preceded the diagnosis of aPAP, with a median duration of 200 days and ranging from 28 to 1,486 days. In 2 cases, corticosteroid therapy was started at 630 days and 3,650 days after the diagnosis of aPAP. The male/female ratio and the median age at diagnosis were somewhat different from those of the large cohort study in 2008 by Inoue *et al.*, whereas the data concerning smoking status and history of dust exposure were similar [8]. At the initiation of corticosteroid therapy, 11, 7, 5, and 1 cases were complicated by hypertension, hyperlipidemia, diabetes mellitus, pulmonary fibrosis, and pneumonia, respectively.

**Table 1:** Demographic data for the present and literature cases

Characteristics	Present cases		Literature data *
	At start of steroid therapy	At the diagnosis	At the diagnosis
Subjects n (range)	31	29 ^#^	223
Age, median yrs	66 (9-82)	64 (9-82)	50 (9-89)
Gender n (%)			
Males	17 (55)	16 (55)	151 (68)
Females	14 (45)	13 (45)	72 (32)
Clinical symptoms n (%)			
Asymptomatic	9 (29)	5 (17)	70 (32)
Symptomatic	22 (71)	24 (83)	150 (68)
Dyspnea	12 (39)	21 (72)	119 (79)
Cough	14 (45)	12 (41)	51 (34)
Sputum	1 (3)	1 (3)	8 (5)
Other	4 (13)	4 (14)	9 (6)
Smoking habits n (%)			
Never-smoker	16 (52)	15 (52)	93 (43)
Current or ex-smoker	15 (48)	14 (48)	124 (57)
Dust exposure n (%)			
Yes	8 (26)	7 (24)	52 (26)
No	23 (74)	22 (76)	147 (74)
Complications n (%)			
None	5 (16)	5 (17)	137 (65)
Hypertension	11 (35)	11 (38)	18 (9)
Infection	0 (0)	5 (17)	12 (6)
Hyperlipidemia	7 (22)	7 (24)	9 (4)
Diabetes mellitus	5 (16)	8 (28)	8 (4)
Autoimmune disorders	0 (0)	0 (0)	3 (1)
Pulmonary fibrosis	2 (6)	1 (3)	3 (1)

*Inoue et al. [8]

^#^Two cases were excluded from enrolled 31 cases, because steroid therapy were started after diagnosis

#### Reasons for corticosteroid therapy

Subjects received corticosteroid therapy for a number of reasons; in 28 cases because the diagnosis suspected initially was other interstitial lung diseases (ILDs), in 2 cases because diagnosis was aPAP complicated by lung fibrosis, and in 1 case, the initial diagnosis was an asthmatic syndrome. Of the 28 cases that were initially diagnosed as other interstitial lung diseases, the diagnoses were IIPs, drug-induced ILD, chronic hypersensitive pneumonitis, chronic eosinophilic pneumonia, and alveolar cell carcinoma in 21, 4, 1, 1, and 1 cases, respectively. In all of these cases, the corticosteroid treatment was tapered and discontinued soon after the diagnosis of aPAP.

#### Dosage and duration of corticosteroid therapy

**Fig. 3:**
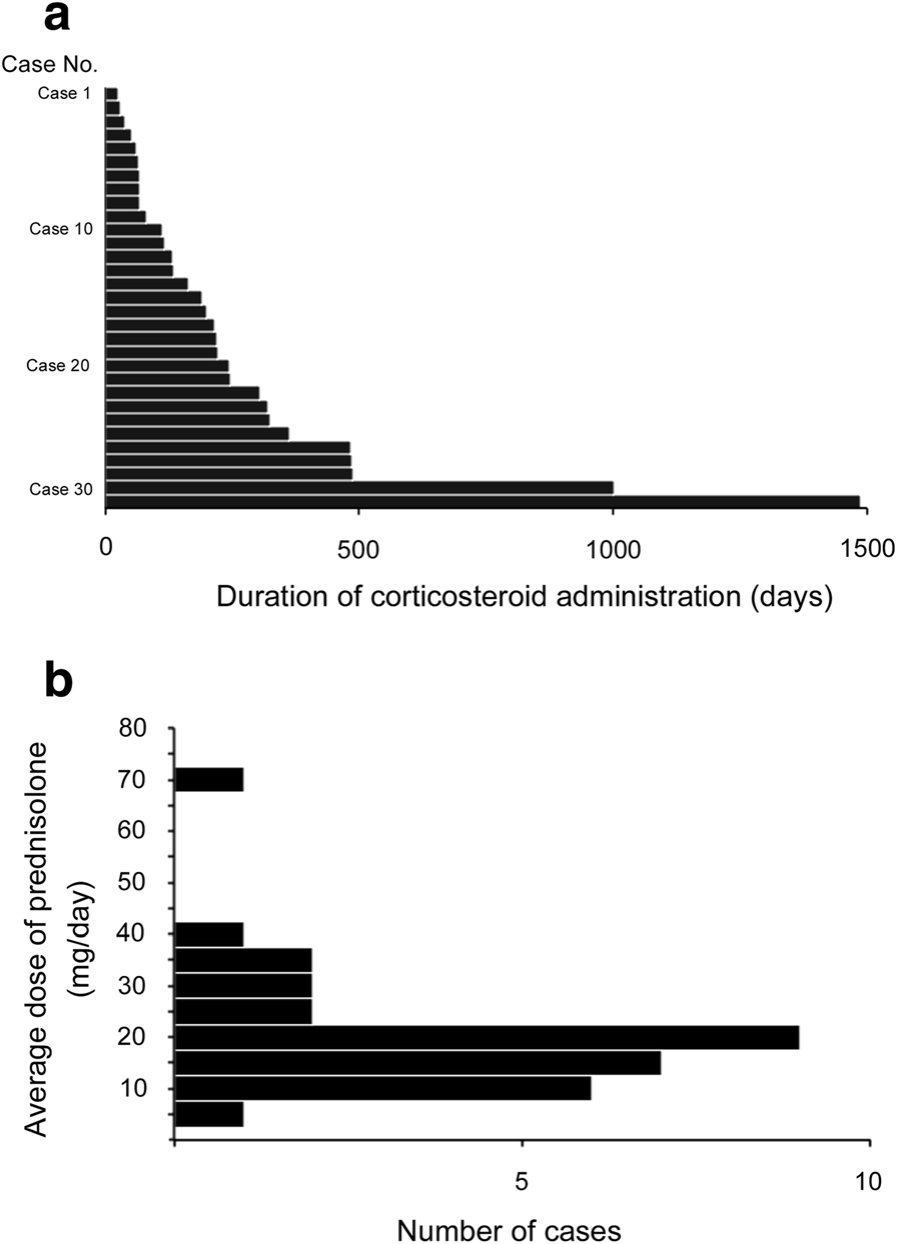
**a** Duration of corticosteroid administration in each case. **b** A histogram for the number of cases that underwent various doses of corticosteroid administration

In all subjects, the cumulative dose of PSL was 400 to 17,780 mg, with a median of 2,750 mg, while the duration ranged 28 to 1,486 days with a median of 191 days (Fig. 3a); 21 cases continued PSL medication for over 90 days. The distribution for the average dose of PSL per day is shown in Fig. 3b. The median daily dose was 18.9 mg/day. When we divided the total cohort into upper and lower halves according to the median PSL value, 16 patients were assigned to the high-dose group, while 15 patients were allocated to the low-dose group. There was no difference in the median age, gender, DSS, smoking history, history of dust exposure, and symptoms before the start of corticosteroid therapy or duration of corticosteroid administration between the two groups (Table 2), demonstrating that the dichotomous process using the median daily dose showed no confounding factor between two groups.

**Table 2:** Difference of background factors or duration of corticosteroid administration between patients received low and high dose corticosteroids

Factors	Statistical methods	P-values
Age	Mann-Whitney U test	0.737
Gender	χ^2^ test	0.200
Disease severity score	χ^2^ test	0.266
Smoking history	χ^2^ test	0.594
Dust exposure history	χ^2^ test	0.916
Dyspnea	χ^2^ test	0.886
Cough	χ^2^ test	0.870
Duration	Mann-Whitney U test	0.114

Background data were collected before the start of corticosteroid therapy. Duration was the serial days of corticosteroid administration. “High” and “low” dose of corticosteroids were described as *Dosage and duration of corticosteroid therapy* in results

Of all 11 cases underwent bolus mPSL (500–1000 mg) administration for 3 days followed by PSL (10–80 mg) for 3 to 24 days. Five repeated the cycles.

#### Clinical course of aPAP during corticosteroid therapy

**Fig. 4:**
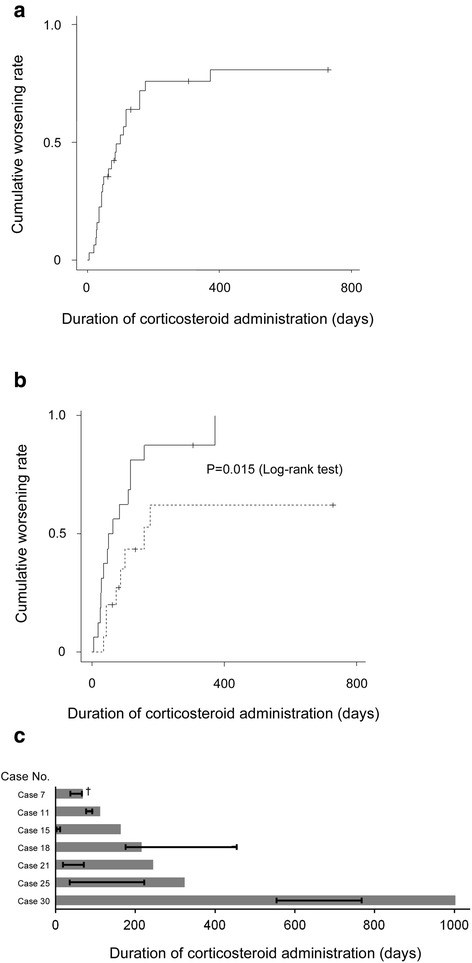
**a** Cumulative rate of worsening estimated using the Kaplan-Meier method. **b** Comparison of the cumulative worsening rate between the high-dose (full-line) and low-dose group (dotted-line). **c** Duration of corticosteroid administration (bar) and antibiotic administration (whisker)

DSS worsened during corticosteroid therapy in 23 cases (74.1 %, Fig. 4a) within 24 (Additional file 1) months and the overall cumulative worsening rate was estimated to be 80.8 % over the observation period, with worsening significantly higher in patients medicated with high-dose corticosteroids than in those with low doses (*p* < 0.02, Fig. 4b). Of those with worsened DSS, 2 died at 39 and 71 days after the start of corticosteroid therapy due to respiratory failure and disseminated aspergillosis, respectively. In 6 patients, infections newly emerged after corticosteroid therapy (*p* < 0.05), including 3 cases of aspergillosis, and 1 case each of nocardiosis, pneumococcal pneumonia, bacterial lung abscess of unknown pathogen, and sepsis of *Pneumococcal pneumoniae* was recorded during corticosteroid therapy, whereas one had before corticosteroid therapy. In 6 of 7 cases, antibiotic administration lasted before the end of corticosteroid therapy (Fig. 4c). It is noteworthy that in 5 cases, DSS improved after the discontinuation of corticosteroid therapy and successful treatment of the infection. In 16 patients with high dose corticosteroid, two patients complicated pulmonary infections during the disease process, of which no patient showed increased DSS after the events of infection. In while, 15 patients with low dose corticosteroid, 4 patients complicated pulmonary infections, of which one patient accompanied increased DSS. On the other hand, in 25 patients after corticosteroids withdrawal, one patient was complicated with pulmonary infections, but none accompanied increase in DSS. As a whole, we consider that increase in DSS is not due to pulmonary infections but mainly due to exacerbation of PAP *per se*.

Consistent with the change in DSS, the number of cases with dyspnea on exertion increased from 5 to 12 cases within the first 3 months of corticosteroid therapy, whereas the number of cases with coughing decreased from 6 to 5 cases. The median serum KL-6 increased from 5033 IU/mL to 7990 IU/mL in 19 cases tested in the initial 3 months (*p* < 0.05).

#### Prognosis after discontinuation of corticosteroid therapy

Of the 27 cases that discontinued corticosteroid therapy after the diagnosis of aPAP, 2 could not be followed-up due to death. Of the other 25 cases, DSS improved in 20 of 25 and 20 of 23 cases in the 6- and 12-month follow-up surveys, respectively. Other 5 and 3 cases in the 6- and 12-month did not change in DSS. Of these 25 cases, 11 cases did not undergo therapeutic intervention but 7 of them improved within 6 months. These results suggest that the deterioration in DSS could be reversible by the discontinuation of corticosteroid therapy.

#### GMAb levels after corticosteroid therapy

In all 29 cases in which the start of corticosteroid therapy preceded the recognition of aPAP, the serum GMAb levels were measured at aPAP diagnosis. The median level was 10.68 (1.04–111.26) μg/mL, similar to the levels reported in the 2008 large cohort study by Inoue *et al.*, suggesting that corticosteroid therapy did not change the autoantibody levels [8]. There was no correlation between autoantibody levels and DSS.

## Discussion

This study demonstrates that corticosteroid therapy might worsen the severity of aPAP and increase the risk for infections. Most cases studied had been treated with corticosteroid therapy for lung diseases other than aPAP such as IIPs, and it was discontinued after the diagnosis of aPAP. Considering that some patients with diffuse GGO on HRCT were still administered corticosteroids for the treatment of putative IIPs without confirmation by pathological evaluation, we believed that the importance of reporting the outcome of corticosteroid therapy in patients with aPAP outweighed the analytical limitations due to methodological difficulties in data collection. Moreover, although our PAP research group has promoted the use of new nomenclature—“autoimmune PAP” instead of “idiopathic PAP”—since 2008, we are concerned that this new nomenclature might lead pulmonary physicians to choose corticosteroid therapy when it is actually not the ideal treatment strategy [8].

Historically, corticosteroids have been used for the treatment of PAP, regardless of whether it is aPAP or secondary PAP [32, 33]. Besides, side effects of corticosteroid was cautioned in a case of hereditary PAP with homozygous stop mutation p. Ser25X of the GMCSF receptor alpha chain [34]. Thus, corticosteroid therapy may exacerbate the alveolar macrophage dysfunction in the above cases. For secondary PAP complicated by myelodysplastic syndrome, we recently reported that use of corticosteroid therapy significantly worsens the survival rate of patients after the diagnosis of PAP [35]. To our knowledge, however, there have been no reports statistically analyzing the outcome of aPAP patients who had been treated with corticosteroid therapy.

As we have a database of large cross sectional study [8], we first tried to set a control group. However, the background factors were so multiple and complicated such as gender, age, duration of observation periods, smoking status, history of dust exposure, history of treatment, and disease activity. Therefore, we realized that it was not so easy to set the control group without selection bias. As often noted by other retrospective studies, researchers must take due care to avoid selection bias of study subjects. Therefore, in this study, as we did not set a control cohort, we compared the worsening rate of the low- and high-dose corticosteroid groups to examine the effect of corticosteroid therapy on the DSS of patients with aPAP. The worsening rate was higher in the latter group than that in the former. Furthermore, we confirmed that there were no confounding factors associated with the background of these two groups. Thus, we concluded that corticosteroid therapy worsened the DSS in aPAP in a dose-dependent manner.

Considering that the disease severity increased in most cases within one year of the start of corticosteroid therapy, dysfunction of the alveolar macrophages progressed in the administrated patients. Although GMAb is the causative agent for aPAP, corticosteroids should be prescribed carefully if for the purpose of reducing the antibody levels, despite being frequently used in other autoimmune diseases. For this purpose, treatments that do not affect alveolar macrophage function but target B cell function or reduction of GMAb concentration, for example anti-CD20 antibody and plasmapheresis, would be ideal [36–38].

The frequency of infections in the present survey, i.e., 7 of 31 cases (22.6 %), was higher than expected, particularly in comparison to our previous cohort study (12 of 212 cases, 5.2 %), although the latter data was from a cross sectional study [8]. Some cases required several months to eliminate their severe infections, and one patient even developed disseminated aspergillosis that proved fatal. Most treating physicians might not pay attention to the side effects of corticosteroid therapy due to the paucity of available information concerning the management of aPAP. In this respect, Punatar *et al.* demonstrated in their meta-analysis that 75 cases of acquired PAP reported between 1950 and 2010 were complicated by opportunistic infections, with overall survival being 56 % [26]. Of those, 13 cases had been treated with long-term corticosteroid therapy.

Notably, 5 of 7 cases complicated by infections during corticosteroid therapy improved, not only in terms of the infection but also the aPAP itself, after the discontinuation of corticosteroid therapy and antibiotic therapy. These curious phenomena are consistent with our clinical experiences and several previous case reports [39–41]. As the number of infected cases were limited and we could not exclude that corticosteroids induced improvement of PAP, we should be careful to interpret these phenomena.

In this study, 28 of 31 patients were initially assumed as other lung diseases such as IIPs, drug-induced ILD, and corticosteroid therapy was prescribed for the treatment of these diseases after clinical of radiological diagnosis based on HRCT without pathological diagnosis. As the HRCT appearance of these diseases and/or clinical features are sometimes indistinguishable from that of aPAP, the present study cautions pulmonary physicians about the casual use of corticosteroids in the absence of a definitive diagnosis by lung biopsy. If corticosteroid therapy is needed to medicate in order to control complex inflammatory diseases (e.g., rheumatoid arthritis), we should extensively survey for latent infections before beginning corticosteroid therapy and carefully monitor for overt infections after corticosteroid therapy initiation. Moreover, the dose should be kept as low as possible.

## Conclusions

This is the first systematic study of patients with aPAP being treated with corticosteroids. Corticosteroid therapy may worsen the DSS in aPAP patients, increasing the risk of infections. We believe that this study will contribute to improved management of aPAP.

## Additional file

Additional file 1: Table S1. Disease severity score (DSS) for each case of pre- and post- start of the corticosteroids administration. (PDF 28 kb)

## Abbreviations

aPAP: autoimmune pulmonary alveolar proteinosis
CSA: Corticosteroid administration
DSS: Disease severity score
GGO: Ground-glass opacity
GMAb: Granulocyte macrophage colony-stimulating factor autoantibody
GM-CSF: Granulocyte macrophage colony-stimulating factor
mPSL: Methylprednisolone
PAP: Pulmonary alveolar proteinosis
PSL: Prednisolone
