# Supplementary material for: Outcome of corticosteroid administration in autoimmune pulmonary alveolar proteinosis: a retrospective cohort study
Source: BMC Pulm Med. 2015 Aug 12;15:88. doi: 10.1186/s12890-015-0085-0 (PMC4534060; doi:10.1186/s12890-015-0085-0)
Supplement: Additional file 1: Table S1. — Disease severity score (DSS) for each case of pre- and post- start of the corticosteroids administration. (PDF 28 kb) [file 12890_2015_85_MOESM1_ESM.pdf]

Supplemental Table 1
Disease severity score (DSS) for each case of pre- and post- start of the corticosteroids administration.

Case No.	Duration (mo.)	Disease severity score (DSS)							
		Pre.	1 mo.(0.5-1.5 mo.)	2 mo.(1.5-2.5 mo.)	3 mo.(2.5-4.5 mo.)	6 mo.(4.5-7.5 mo.)	12 mo.(9-15 mo.)	18 mo.(15-21 mo.)	24 mo.(21-27 mo.)
1	0.82	2	3	5	2 *	1	1	1	N/A
2	0.99	3	4	5	5	3	2	1	1
3	1.28	4	5	†	-	-	-	-	-
4	1.71	3	4	4 *	5	5	2	2	N/A
5	2.04	2	3	5	N/A	3	1	1	1
6	2.17	1	1	4	1	1	1	1	1
7	2.27	2	2	2	2	N/A	N/A	N/A	N/A
8	2.27	2	2	5	†	-	-	-	-
9	2.27	3	5	5 #	3 #	1 #	1	1	1
10	2.70	1	1	1	4	1	1	1	1
11	3.88	1	1	1	1	1	1	1	1
12	4.31	2	2	2	3	2	2	1	1
13	4.41	1	1	1	1	2	2	N/A	N/A
14	4.47	4	5	5 *	3	1	1	1	1
15	5.39	4	4	N/A	3	3 #	1	3	N/A
16	6.28	3	N/A	N/A	N/A	4 *	1	N/A	N/A
17	6.58	3	N/A	N/A	4 *	3	2	2	3
18	7.10	1	1	1	3	3	2	2	2
19	7.23	2	3	3	3 *	2	1	1	N/A
20	7.33	2	2	2	4	3 *	1	1	2
21	8.05	5	5	5	4	4	1	1	1
22	8.12	1	1	1	1	2	1	1	1
23	9.07	4	2	1	2	4	5 *	2	2
24	10.06	1	1	1	1	1	3 *	2	2
25	10.52	2	2	N/A	2	3	5 *	1	1
26	11.90	2	2	3	N/A	4	5	N/A	N/A
27	15.91	1	1	1	1	1	3	2	1
28	16.01	2	3	3	3	5	5	5	N/A
29	16.08	2	2	2	2	2	2	2	2
30	32.98	1	1	1	1	1	1	1	1
31	48.85	2	2	2	2	2	2	2	2

mo.: month; Pre.: Pre-administration of corticosteroids; *: Whole lung lavage;
#: Inhalation of granulocyte/macrophage colony-stimulating factor; N/A: Not available; †: deceased.
An area indicated by the blue shows duration of corticosteroids administration.
